# Iron Carbonate Mineral Waters without Excess of Carbonic Acid

**Published:** 1918-10

**Authors:** Felix Von Oefele

**Affiliations:** New York City


### Iron Carbonate Mineral Waters Without Excess of Carbonic Acid.

DR. FELIX VON OEFELE, New York.

Iron is what we called in previous publications a valid constituent of mineral waters. The element iron is a predominant constituent of the whole mass of the earth. It seems to be the principal part of the interior of the earth. Of the surface strata it forms a smaller percentage on account of its high specific gravity sinking in to the depth. The different oxydes of iron are soluble in water with great difficulty. The soluble iron salts derived from the different iron oxydes show a remarkable tendency to dissociate with a precipitation of oxydes or basic salts. Where strong acids are present in excess a high percentage of iron salts may be dissolved. These mineral waters are listed with the respective acids within the superminerализed waters.

Of the carbon derivatives tartaric acid, citric acid, oxalic acid and many other multiple organic acids form soluble iron compounds of the ferrous as well as of the ferric type. The iron carbonate is only the lowest member of this group.

Iron carbonate mineral waters are low mineralized waters. The organic constituents of the drift in the oxydation degree of crenic acid are able to react to iron salts of the soil and to form a solution. The further oxydation will form ferrocarbonate. Ferrous carbonate is soluble in an excess of carbonic acid. Every mineral spring contains some small trace of this ferrous bicarbonate dissolved, many contain determinable amount. These waters having appeared to the surface give off the excess of carbonic acid, absorb oxygen of the air and precipitate insoluble black iron oxydul oxyde. In this way the black magnetic iron stone is formed having lost the water. Where the oxydation advances the brown iron stone may be formed as semihydrated product and having lost the hydration entirely, the red iron stone is formed. Where the ferrous carbonate is precipitated by evaporation of the carbonic acid excess and where the precipitate so formed is protected from further oxydation, the iron spar is formed. Where a sufficient excess of carbonic acid to form a ferrous bicarbonate is present and persists for some time, an iron carbonate mineral water appears which we will call steel water.

In as much as steel is a combination of iron and carbon, iron carbonate springs not considering the oxygen of the molecules present have been called steel springs or with a scientific foreign word chalpbeate springs.

I have collected a list of 79 German mineral spring locations known by iron content of their mineral springs.

SIMPLE STEEL SPRINGS.

The simple steel springs are as a rule low mineralized drift springs or shallow strata springs. The dissolved carbon dioxyde under high atmospheric pressure increases the dissociating and dissolving power more for lime and magnesia than for iron salts. Springs with carbonic acid of very deep origin contain mostly iron as a small and not remarkable percentage of total solids. The carbon dioxyde of drift springs is a product of organic decomposition within the drift.

Iron is found over the entire earth's surface. A sufficient concentration of iron to form iron springs is very irregularly arranged in sedimentary rocks. Simple steel springs may be expected as isolated springs in all the sedimentary rocks furnishing low mineralized waters where the oldest central mountains of crystalline rocks show the mica contained rich in iron.

The condition for thermal steel springs is very rare. Where low mineralized thermal water rises from deeper rocks, the condition of the carbonic acid of the drift loses the possibilities to dissolve iron salts of the uppermost strata. Simple steel springs are mostly cold springs.

What we will call abundant iron content of a mineral spring exceeds rarely 0.001 to 0.01% iron salts calculated to the water itself. Many of the iron springs contain usually an additional small amount of arsen. If arsen reaches over a certain percentage of iron, the spring may be called an arsen spring.

The additional presence of a small amount of phosphoric acid and silicic acid in a mineral spring keeps the oxydation products of iron for some time in a semi-dissolved opalescent condition. The time necessary for precipitation depends from the amount of sulphuric acid present. A scarcity of sulfates hurries the precipitate to become yellow and later ocker-colored. Other conditions change the colors.

Steel springs form a yellow to brown or red sediment, the so-called spring ocker. It is easily noticed by every layman. This precipitate is known every where in the old world as iron compound. Steel springs are long used mineral springs known in a time long before analytical chemistry. Pyrmont in Germany was used in the times before the Roman empire. Old northern fibulas with characteristic decorations have been found as token of thankful patients.

For the modern scientist, the precipitation of spring ocker is not a complete evidence of a real iron spring. Other constituents may prevail. This prevalence may be so strong that the iron present is not remarkable for the therapeutic value and is not noticed in the classification of many mineral springs. In these cases often the principal amount of iron compounds is precipitated. Arsen is completely precipitated. Other constituents become insoluble only in small traces or not at all. The spring ocker is a concentrate of the spring iron and related substances. The water remaining is poorer in these constituents.

The tendency to form spring ocker makes it hard for bottling and shipping the related waters. It is worse for waters where the iron is present in very small amount and is not remarkable for the therapeutic value of the water concerned. Many such mineral waters are found in America where the iron contained is considered more as an impurity than as a therapeutic adjuvant. I found the trail of Professor Chandler who recommended at Saratoga Springs, at Ballston Spa and elsewhere in many cases the addition of citric acid to overcome the precipitate of troubling iron. This addition is an adulteration if the label bears the claim of natural mineral water.

This is not the place to give my advice and experience for bottling without chemical addition and without precipitate. I wish only to emphasize that bottling is possible. No air whatever should come in contact with iron water until it is used.

In Europe there are springs with iron carbonate without considerable excess of carbonic acid, iron carbonate springs with considerable excess of carbonic acid, alkali or earth carbonate springs with considerable adjutory iron, sodium chloride springs with considerable iron, iron sulfate springs and sodium sulfate springs with considerable iron. The average of American mineral springs is less basic and more acid than in Europe. On this basis America contains additional mineral springs with remarkable amount of iron chloride and acid springs with iron sulfide. Springs containing iron chloride are very rare in Europe.

The leading iron springs of Europe and America are the cold simple steel springs. The indication of all iron springs relates to an increased iron supply in an insufficient function of the blood present.

James K. Crook unites all the mineral waters containing iron into the classification of Chalybeate Springs. He makes some subdivisions. Crook mentions as the most important Chalybeate Springs: Round Spring at Aurora Springs, Missouri; Matchless Mineral Wells, Alabama; Arkansas Lithia Springs, Arkansas; Pacific Congress Springs, Fulton Wells and Pagoda Springs, California; Fuller Springs, Georgia; Indian Springs, Indiana; White Sulphur Springs, Iowa;

Topeka Mineral Wells, Kansas; Addison Springs, Maine; Londonderry Lithia Spring, Vermont; Health House Springs, New Jersey; Adirondack Mineral Springs and Oak Orchard Springs, New York and Putnam Springs at Saratoga Springs, New York; Gaylord Springs and Gulick Springs, Pennsylvania; Austin Springs, Tennessee; Overall Mineral Wells, Texas; Bath Alum Springs and Rock Enon Springs, Virginia.

Not only one of these mineral springs mentioned by Crook can be classified. A sufficient analysis of many of them is missing. Additionally Cienega (Manantiales de la —) in Yucatan, Mexico, is claimed to contain abundant iron. If such and similar other claims are believed at all, they do not enable a classification of the mineral spring concerned.

### SIMPLE IRON SPRINGS.

Springs below 0.1% total solids with over 10% iron salts are real iron springs. They contain the iron as carbonate of the oxydul without a remarkable excess of free carbonic acid. 1 to 10% of total solids iron salts is a remarkable iron content. If hydrochloric or sulfuric acid prevails or carbonic acid reaches a remarkable atmospheric pressure, a higher amount of iron may be present. Such springs will be classified later with these respective acids.

The properties of iron salts are very striking for the human senses. The submineralized and the supermineralized springs containing remarkable amount of iron can not be separated strictly every time from the real iron springs, particularly not for the therapeutic indications.

### THERMAL SIMPLE STEEL SPRINGS.

There are no real thermal iron waters in Germany. Few of them are in Austria, France and Russia.

Of Newsom's Arroyo Grande Springs, California, different springs are rich in iron and potassium. The warmest of 38°C is the only one that has been analysed. 0.0634% total solids with near 11% iron carbonate, 8% potassium salts and 25% magnesium and calcium salts. The water contains 0.0012% free carbonic acid and 0.0004% weight hydrogen sulfide.

Puller Springs, Montana, claims without analysis two iron springs of 35°C and 41°C 1850 meter over sea level, this is claimed for rheumatism.

### COLD SIMPLE STEEL SPRINGS.

All the "Steel waters" of Germany and Austria are cold. Not one exceeds 20°C. The condition above explained producing steel waters is rarely found in plains. It is often found in the valleys of hilly districts having concentrated the iron extracts of the neighbouring rocks. Often the steel springs have a high elevation over sea level.

In many cases where steel water is indicated, this elevation is important for therapeutic efficiency.

European Springs of less than 1000 feet elevation are: Schwalbach in the Taunus, Hessen-Nassau with Stahlbrunnen, Pyrmont, Waldeck with Trinkbrunnen, Brueckenau in the Rhoen Mountains, Driburg and Boklet.

Between 1000 and 2000 feet are; Spaa in Belgium with a spring called "Pouhou," Liebenstein, Meiningen, Petersthal, Cudowa, Altwasser-Flinsberg, Silesia, Franzensbad, Bohemia, Lobenstein, Reuss, Alexisbad in Harz, Elster, Griesbach, Autogast, Rippoldsau, Schwarzwald, Reinerz.

5500 feet elevation shows St. Moritz, Switzerland with "Grand Source."

The elevation as well as the iron absorbed causes a reaction forming a larger number of red blood corpuscles. An excessive high elevation exceeds often the power to react of the patient concerned. For very important cases, it is usual in Europe to select a place 900 to 1200 feet higher than the steady residence for the first stay. After few weeks then a second place of higher elevation is recommended.

The most European Steel Springs are submineralized springs with predominant or remarkable amount of iron. The most European iron spring resorts contain more than one spring. We list only the highest and lowest figures in the following enumeration: St. Moritz 2 to 4% of total solids, Elster 1 to 4%, Pyrmont 1.5 to 3%, Franzensbad 0.2 to 2.5% and Liebenstein 0.5% of total solids iron compounds.

There are no iron springs properly speaking. The absolute amount of iron compounds is very low: Brueckenau 0.0002%, Reinerz 0.001 to 0.004%, Franzensbad 0.001 to 0.013%, St. Moritz 0.003 to 0.005%, Pyrmont 0.004 to 0.008%, Rippoldsau 0.005 to 0.013%, Elster 0.006 to 0.009%, Triburg 0.007% and Liebenstein 0.009%. All kinds of iron waters have been listed above.

European Steel Springs with remarkable iron content of this class are:

Wernazer Quelle at Brueckenau contains 0.01% bicarbonates of the earths, very little other constituents and almost 2% of total solids ferrous bicarbonate.

Freienwalde with Koenigsquelle and Badequelle contains about 0.03% total solids; bicarbonates of earths predominate and carbonic acid is somewhat in excess. 2 to 7% of total solids are ferrous bicarbonate. The advantage of Freienwalde is the situation in the neighborhood of Berlin. The metropolitan people prefer a place for their sick girls and young brides a short distance from the residence.

Kohlgrub, Bavary, contains 0.09% total solids almost exclusively bicarbonates of earths and 7% of total solids ferrous bicarbonate. It may be reached within two hours from Munich.

Other famous European steel springs are:

Tunbridge Wells in England, Liege in Belgium, Grisons in Switzerland, Krynica in Galicia, Syliacs in Hungary and Rodna in Siebenbuergen.

150 to 100 years ago steel waters were highly recommended by every scientific physician. They have been used for about every indication with the sideline of improved blood. These recommendations exceeded evidently the true value. The reaction followed with an insufficient estimation of steel springs today. The truth seems to be between the extremes.

An improvement of the blood by an increased iron supply was the old idea. The time of reaction denied the resorption of iron at all. But this is wrong. My friend Goldman at Sopron, Hungary, showed with scientific exactness the ability of the human skin for absorption of iron to the system. The internal absorption is still greater.

It is explained that the iron percentage of the strongest iron springs is very low. Nevertheless every internal use of iron water forces the digestive tract to hard work. It is less hard than the digestion of artificial iron preparations, but it is hard. It needs an individual selection to recommend an iron spring and an expert physician is necessary as guard to accommodate the single application.

Some times the excess of carbonic acid, some times the low temperature causes trouble. In these cases a certain preparation of the water for the single patient is necessary. Women folks or females are mostly the users of iron springs. The tendency of iron precipitation colors or shades the teeth. This is often feared on a cosmetic reason. At many iron spring resorts it is usual to drink the iron water through straws as prevention of a contact with the teeth. At other springs the mouth is washed immediately after the iron water by iron free water or by suitable artificial preparations.

It is an old experience that raw berries and fruits should not be used during an internal iron spring treatment. The experience is correct, but it is a mistake to blame the tannic acid for the bad effect.

### INDICATIONS FOR IRON SPRINGS

The physiological and therapeutical usefulness of iron springs is based on the small iron content of normal human organism. The total amount of iron of a healthy adult is about 3 grams. In cases of chlorosis the body is deficient in iron. This shortage may amount up from 0.6 to 1.25 grams in serious cases. The daily supply and output is about 0.06 grams. The absolute need of iron is understood to be very small. But the amount of iron supply is less important than the possibility of absorption of an iron supply and of retention of the iron absorbed. Concentrated anorganic iron compounds are often easily absorbed within the upper part of digestive tract and after a few hours excreted by the colon without real assimilation.

The usual medical text-books do not show a clear idea what happens with steel waters within the digestive tract. Our own very numerous examinations with single duodenal tubes or with combination of stomach tube and duodenal tube prove the superiority of submineralized steel springs. Food and high concentrated iron drugs stay for a long time in the stomach and react chemically with the free hydrochloric acid of the stomach. Iron concerned becomes iron chloride which is absorbed and immediately reexcreted by the colon. Submineralized steel water enters and passes the empty stomach without delay and without production or influence of free hydrochloric acid. It enters as ferrous bicarbonate the small intestines and reaches the blood as ferrous bicarbonate which is able to enter the blood corpuscles while ferrous chloride and ferric chloride are not able.

This is in accordance with the general experience of medical practitioners that the prescription of a natural iron spring is more efficacious than any artificial iron preparation. Since the powdered haematite of old Babylonians up to the most recent preparations, history repeats that a new artificial iron compound comes in use, becomes recommended with exaggeractions and becomes finally antiquated and forgotten. That opens a place for new iron preparations repeating the same history. The only persistant iron therapy through thousands and thousands of years in the old country is the use of iron springs. Pyrmont in Germany has been already mentioned and used according to excavations more than two thousand years ago by a population of its vicinity not far over the culture of Indians at the time of Columbus and is still used by the patients of international high life of today.

Iron given by the mouth has been found in the lymphe of the ductus thoracicus. Iron chlorides and their derivatives disturb there the regular chemism of Syntonines, Peptones and Aminoacids. This circumstance forces the automatic regulation of the organism to a quick excretion of the total iron chloride absorbed.

If iron is taken by the mouth, a part stays unabsorbed within the digestive tract and is excreted by the feces. The feces become black-green. The excreted iron compound concerned is iron oxydul according to Quincke. Some decades ago it was mistaken for iron sulfide. Usefully absorbed iron has to remain a long time within the organism in organic combinations. It is important that the blackish green feces are not seen in iron mineral spring resorts after the use of iron mineral spring waters. It happens there only as an exception if the iron water had been taken together with fresh berries.

The principal amount of iron of the human body is contained in the haemoglobin. The haemoglobin has the property in an excess of carbonic acid to combine with carbonic acid and in an excess of oxygen to combine with oxygen. This circumstance enables the haemoglobin to take the excess of carbonic acid formed in the tissues and to excrete by the lungs and to join within the lungs some of the excess of oxygen of the air and to carry to the tissues.

The organism has to intend to keep the stock of haemoglobin. Inevitably some of the haemoglobin of the blood is used up and has to be replaced by haemoglobin newly formed. That is where the principal amount of new iron supply of the body is for. Different races and different individuals of the human beings require different amounts of iron used up in the metabolism. The same human being needs less haemoglobin staying on sealevel plains where the air shows an average higher barometric pressure and needs more haemoglobin far up in the mountains with very thin air. This is easily understood by the above explained work of haemoglobin as oxygen and carbon dioxyde carrier. Every human organism shows a certain adaptation for regulation of the haemoglobin present. We spoke above about this matter calling it the power to react.

Besides the tendency to stay within the level of haemoglobin percentage liver, spleen and marrow are places of storage for iron excess for periods of increased need. The physiological functions of the female organism shows larger undulations of iron content than the male. It is understood therefore that female patients need oftener a therapeutic increase of iron supply and that a high elevation over sea level favors the assimilation of a higher iron supply. For no other class of springs the high elevation over sea level is equally important.

Chlorosis, anaemic conditions after serious losses of blood, convalescence from acute diseases, overwork of muscles or brain, Basedows disease, pathologic condition of spleen, liver or kidneys after malaria or salvarsan treatment, chronic diseases of the female sexual organs, irregular menstruation, chronic metritis, sterility, habitual abortion, pathological feebleness of the male sexual organs, particularly spermatorrhoea, pollution, impotency, chronic disease of the nervous system, general functional neurosis, nervosity, neurasthenia, hysteria, spinal irritation are the indications for the internal use of steel springs. The female patients predominate.

Where iron waters and especially iron carbonate waters are in contact with the air, oxygen of the air is absorbed and a colored precipitate is formed as explained above. This direct precipitation without other natural admixture we will call "spring ocker."

This spring ocker collected at the mineral spring itself may be used for internal treatment. It is a natural finely divided and resorbable iron powder with a slight arsen reinforcement. It is the best internal product which may be transported from an iron spring without necessary deterioration.

The author has prescribed successfully in his European practice daily 0.01 gram increasing in 10 days to 0.1 gram and again decreasing to 0.01 gram spring ocker, with sugar mixed and compressed to tablets in cases of chlorosis of girls. It is the best obtainable iron preparation in a dry shape. In regard to the effect on the skin color of girls, Bombelon called this preparation beautifying lozenges.

Where the iron water runs from the springs and becomes mixed with decayed organic matter, particularly swamp grass and tree leaves, it forms black, red or yellow iron mud. This mud is very valuable for external treatment. We will call it "steel moor." It is used for general mud baths and for local mud cataplasms.

Steel moor is abundantly used in European mineral spring places. This use is somewhat restricted as far as many mineral spring places became entirely exhausted of the prehistoric deposits of this material. Other places use only a restricted amount to evade this exhaustion.

This original natural "steel moor" must be dug from the moist soil and piled up. It becomes dry and is decayed further by atmospheric air. For the application it is mixed with hot water or boiled. A thick heavy liquid may be produced for bathing in. Many kinds of European "steel moor" contain sulfates. The iron sulfate present and often half compound acids of concerned acid salts have a strong chemical influence to the human skin with a therapeutic efficiency. Steel moor baths may be used with a higher temperature than other mineral baths and common baths. The body does not feel attacked after an overheated mud bath of steel moor.

There have been told certain indications for Akratotherms and almost contrary indications will be told for brine baths and carbonated brine baths. Baths with steel moor are an intermediary link between both. The particular indications are neuralgia, paralysis, chronic muscular and articular rheumatism, arthritic deposits, the following conditions from traumatic influences are exudates of the pelvic cavity.

If we travel up hills from the eastern sea shore, we very soon find many iron compounds in some places of glacial drift. New Jersey is known as the state of the red mud what means really mud rich in iron. The northwestern corner of

Long Island contains waters rich in iron. The insufficient elevation and other less desirable properties excluded the springs of these locations from the medicinal springs. They have been also continuously excluded from the water supply of greater New York for domestic use and for industrial use. They are found where cretaceous rocks are covered by glacial drift. Valuable iron springs are situated in different places of the Atlantic mountain declines farther up.

### AMERICAN LOW MINERALIZED COLD IRON WATERS.

Sparta Magnetic Well at Sparta, Monroe County, Wisconsin, 0.033% total solids including over 60% iron carbonate.

Chalybeate Spa at White Sulphur Springs, Sullivan County, New York, with 0.0068% total solids including over 50% iron salts. The author has personally analysed and studied this mineral spring place. Accommodations for high class people are found at White Sulphur Spring House. Many cheaper houses are a short distance away; cottages can be rented by the week for housekeeping. The spring is in use since 1888 and is equipped with a bathing house. Iron mud of inexhaustible amount is present. The mineral springs themselves are not yet sufficiently accessible and not yet properly collected. The place is filled during July and August. The therapeutical conditions for gynaecological diseases are far more favorable from middle of May to July. It is the nearest iron spring for New York city and joins the advantage of Freienwalde in regard to Berlin with the medicinal properties of St. Moritz, Switzerland.

Aurora Springs, Missouri, with 0.0357% total solids including over 30% iron salts.

Iron Lithia Springs, Virginia, with 0.0380% total solids including 23% iron sulfate.

Hurlburts Springs at Cambridge Springs, Pennsylvania, with 0.0088% total solids including 10% iron oxyde.

Remarkable amount of iron is contained in the following mineral springs:

Adirondack Mineral Spring, Washington County, New York, with 0.1307% total solids can not be separated from this group. It is situated in the sixth district of the Middle Atlantic Area as a northern outpost island of the Catskill group of medicinal springs. The total solids include 61% earthy salts with predominant carbonates and about 7% iron carbonate equal to 0.0086% of the water itself.

Chalybeate Spring at Harbin Hot Springs, Lake County, California, with 0.0791% total solids including nearly 70% alkali salts and 4% ferrous carbonate.

Chalybeate Spring at Estill Springs, Estill County, Kentucky, with 0.0882% total solids including 75% earthy salts and 3.5% iron carbonate. Half combined and free carbonic acid are present in 0.112% weight of water.

Claremonde Chalybeate Spring, Washington County, Georgia, containing about 0.0007% iron carbonate in the water and few other contents not determined quantitatively.

Green Lawn Springs, Jefferson County, Illinois, P. O. Mount Vernon, claims six springs from a vertical stratum of slate. One is called Washington Spring containing carbonates of iron and earths and excess of sulfohydrogen and carbonic acid gas. The water is internally used for dyspepsia, torpid liver, biliousness and other disorders of the chylopoetic organs. The bathing with this water is claimed useful for rheumatism.

Chalybeate Spring at Greenbrier White Sulphur Springs, Greenbrier County, West Virginia, with carbonate of iron has not been fully analysed.

Mountain Springs, Lancaster County, Pennsylvania, may be a carbonated iron water with high elevation.

North Haven Pool, New Haven County, Connecticut, contains iron carbonate and other contents, only qualitatively determined.

Bartlett Mineral Spring with 0.0230% total solids and Petticord Spring 0.0189% total solids at Cambridge Springs, Pennsylvania, contain remarkable amount of iron.

---

**Extradorsal Anaesthesia.** Gil Vernet, Hojas Medicas, July 1917, injects to both sides of the sacral trunk, in the shape of a V. The solutions consists of novocaine 0.20, potassium sulphate 0.20, paraphenol disulphonate of anaesthesin 0.20, adrenalin hydrochlorate, 1:1000, 1 c.c., sodium chlorid, 1: 1000, 0.9 c.c. The patient is placed on the belly, with the pelvis slightly elevated. A needle 12 c.m. long is used. Anaesthesia results in 20-30 minutes and lasts 1-2 hours. Colporraphy, internal and external urethrotomy, haemorrhoid operations, prostatectomies, castrations and operations for rectal prolapse, have been successfully performed.

---

**Prolonged Gestation.** Roger Williams, of London, Practitioner, Jan., reports a case delivered of a healthy female child, by forceps. The last menstruation ceased exactly one year previously, the last coitus having preceded it a few days.

---

**Ether as an Antiseptic.** Le Moniteur Med., Mch. 19, states that military experience has shown this substance to prevent suppuration and gangrene and to facilitate rapid healing. One surgeon even relates a case that healed rapidly after a bite by a rabid dromedary.

